# The need to accelerate COVID-19 education across medical schools

**DOI:** 10.5116/ijme.6488.1a6e

**Published:** 2023-06-16

**Authors:** Susan J. Rees, Nada Hamad

**Affiliations:** 1 School of Clinical Medicine, Faculty of Medicine and Health, University of New South Wales, Australia

**Keywords:** Curriculum development, COVID-19, disease management, health equity

## Introduction

COVID-19 (SARS-CoV-2) is one of the most devastating viral outbreaks of modern history, with serious impacts on medical systems and clinical practice. Despite evidence that medical practice is slowly adapting to manage the considerable health effects of COVID-19, many teaching environments have not formally integrated COVID-19 into their curriculum.^1^^-^^3^  There is a need for COVID-19 specific education and curriculum development, and for the evolving scientific knowledge base to be shared globally across medical schools.^4^ This is because the magnitude of illness and risk for mortality associated with the novel COVID-19 virus requires that all students have the capacity to integrate COVID-19 learning into their clinical practice and reasoning, decision making, and patient management.^1^^-^^3^

### COVID-19 Disease and Disability

The disease emerged in late 2019 and was declared by the World Health Organisation to be a pandemic on March 11, 2020. There is now compelling evidence that COVID-19 can have sequelae in multiple organ systems, including cardiovascular and brain diseases.^2^^,^^5^ The research shows an increased risk for serious illness and persisting disability, even in younger people without prior underlying health issues.^2^^,^^3^ COVID-19 is also an exemplar of social, cultural economic determinants of health, and how inequity influences health outcomes. The pandemic offers salient lessons for students in understanding how bias, ethnicity, and geographical location, for example, can increase risk of survival or missed diagnosis of post COVID sequelae.^6^

Despite COVID-19’s manifest relationship to medicine and the social determinants of health, the emphasis in most teaching programs and the published literature has been on the functional challenges of teaching safely and efficiently during the pandemic, focusing on hybrid and electronic teaching methods.^4^^,^^7^ COVID-19 is, however, not only a challenge to teaching processes; it is radically shaping human health and functioning at a population level. Our role as medical educators is to keep abreast of the changing scientific evidence, anticipate what students need to know, and ensure they have the skills required to integrate rapidly evolving evidence and understand how it impacts practice.^4^^,^^8^^,^^9^

### Informing the COVID-19 Curriculum

Continuity in learning related to this new and evolving disease is required across medical schools. One mechanism to advance national and international uniformity with education is to share resources and enhance communication across medical schools.^4^^,^^8^ Suggestions for enhancing communication include the use of an evidence-informed global repository for educators to access.^8^ Resources should include contemporary articles and materials linked with relevant areas of medical curricula. The unpublished Medical Student COVID-19 curriculum by and for students has made significant inroads into building an evidence base.^9^

We have identified some key thematic areas in curricula that should include a COVID-19 focus. Although courses are not universally standardised, most include a study of anatomy and physiology, as well as the foundations of disease and microbiology. Our recommendation is to include the bio-physiology and pathogenesis of COVID-19 as well as ‘Long COVID’ and their relationship with disease diagnosis, immunity, treatment, and prevention. The study of clinical skills includes history-taking, where students explore COVID-19 related diseases such as mental health and cardiovascular symptoms.^1^^,^^2^ Intersectional factors are highly relevant to COVID-19, including how social inequalities shaped by race, gender, place, and socio-economic status may be relevant to the patient’s presenting illness, risk, and recovery. Courses that involve the study of community and preventative medicine should include a focus on the interaction between COVID-19 and the social determinants health, including, for example, how it shapes access to vaccination and anti-viral medications, co-morbid chronic illness, domestic violence, and access to health care.

The study of medicine commonly targets unique life stages, including paediatrics and geriatrics. These curricula should now include the impact of COVID-19 on health across all stages of life, including childhood, adolescence, pregnancy, and old age. Courses focusing on epidemiology and biostatistics should include COVID-19, for example, incidence and epidemiological waves, Long COVID, and the study of excess deaths. These changes in populational level health should be linked to public health policies and practices to prevent and respond to COVID-19.

The contemporary study of psychiatry and neurology should emphasise mental illness and neurological diseases linked to COVID-19, as well as the impact of related socio-economic stress on new onset and pre-existing mental illness and psychological wellbeing.^5^ Finally, all curricula should teach students the importance of self-care, particularly in times of significant health system stress.

In summary, COVID-19 has disrupted health systems, undermined public health status, driven down life expectancy, and impaired quality of life.^10^ The virus continues to mutate, with devastating and multiple impacts on human health. All medical curricula must reflect the significance of COVID-19 to clinical practice and human health. Medical students are being trained at an extraordinary time in the history of human disease and wellbeing, and the medical curriculum must equip them to be at the forefront of understanding and shaping of medical practice.

### Acknowledgements

We wish to thank Batool Moussa BA., LLB., M.SocPol. for reading and editing this manuscript.

### Conflict of Interest

The authors declare that they have no conflict of interest.
